# The high protein expression of FOXO3, but not that of FOXO1, is associated with markers of good prognosis

**DOI:** 10.1038/s41598-020-63895-8

**Published:** 2020-04-24

**Authors:** François Lallemand, Sophie Vacher, Leanne de Koning, Ambre Petitalot, Adrien Briaux, Keltouma Driouch, Céline Callens, Anne Schnitzler, Caroline Lecerf, Floriane Oulie-Bard, Aurélie Barbet, Anne Vincent, Sophie Zinn-Justin, Bernard S. Lopez, Rosette Lidereau, Ivan Bieche, Sandrine M. Caputo

**Affiliations:** 1 Service de génétique, unité de pharmacogénomique, Institut Curie, 26 rue d’Ulm, Paris, France; 2 Paris Sciences Lettres Research University, Paris, France; 3 Translational Research Department, Institut Curie, PSL Research University, 26 rue d’Ulm, Paris, France; 4 Service de génétique, unité de génétique constitutionnelle, Institut Curie, 26 rue d’Ulm, Paris, France; 5 CNRS UMR 8200, Gustave Roussy Cancer Institute, Université Paris-Saclay, équipe labélisée par la Ligue contre le cancer, Villejuif, France; 6 Institut Cochin, INSERM U1016, UMR 8104 CNRS, Université de Paris, 75014 Paris, France; 7 Centre de Recherche en Cancérologie de Lyon (CRCL)/INSERM U1052-CNRS UMR5286 Centre Léon Bérard, 28 Rue Laënnec, 69373 Cedex 08, Lyon, France., Lyon, France; 8 Laboratoire de Biologie Structurale et Radiobiologie, Institute for Integrative Biology of the Cell (CEA, CNRS, University Paris South), University Paris-Saclay, Gif-sur-Yvette, France; 9 INSERM U1016, Université Paris Descartes, 4 avenue de l’observatoire, Paris, France

**Keywords:** Cancer genetics, Prognostic markers

## Abstract

To better define the role of FOXO1 and FOXO3 transcriptional factors in breast carcinogenesis, we performed a comparative study of their expression at both the RNA and protein levels in a series of human breast tumors. We used qRT-PCR assay to quantify mRNA expression and Reverse Phase Protein Arrays (RPPA) to quantify protein expression in 218 breast tumors from patients with known clinical/pathological status and outcome. Weak correlations were observed between mRNA and protein expressions for both *FOXO1* and *FOXO*3 genes. High expression of FOXO3 protein, but not FOXO1 protein, was a good prognostic marker, negatively correlated with KI67 and markers of activity of the PI3K/AKT/mTOR oncogenic pathway, and positively correlated with p53, a marker of apoptosis. Moreover, FOXO3 protein expression, but not FOXO1 protein expression, was also negatively correlated with various proteins involved in different DNA repair mechanisms. FOXO3 protein, but not FOXO1 protein, appears to be a tumor suppressor that inhibits breast cancer by altering DNA damage response (DDR), thereby inducing p53-dependent apoptosis. This antitumor effect appears to be suppressed by excessive activity of the PI3K/AKT/mTOR pathway. High FOXO3 protein expression could be a biomarker of deficient DDR in breast tumors.

## Introduction

Breast cancer is the most common solid malignancy in women in both developed and developing countries^1^. Based on gene expression profiling, this pathology have been classified into four subtypes: luminal, human epidermal growth factor receptor 2 (HER2/ERBB2)-enriched, basal-like and normal-like^2^. Most breast tumors of the basal-like subtype are triple-negative (TN), which means that they do not express estrogen and progesterone receptors, and lack ERBB2 overexpression^3^.

The forkhead box O (FOXO) family is a subclass of the forkhead family of transcription factors and consists of four members: FOXO1, FOXO3, FOXO4 and FOXO6^4^. These four proteins possess the conserved DNA-binding domain named forkhead domain or winged-helix domain^5,6^. They are involved in the regulation of various cellular processes such as cell cycle, apoptosis, metabolism, and DNA repair^4,7,8^. Their transcriptional activity is modulated notably by acetylation, ubiquitination, and phosphorylation^5,9^. Once activated, the PI3K/AKT/mTOR cellular pathway induces the phosphorylation of FOXO proteins by AKT. This phosphorylation leads to the exclusion of FOXO proteins from the nucleus, inhibiting therefore their capacity to modulate the transcription of their target genes^4,10,11^. This phosphorylation has also been shown to promote the degradation of FOXO3 protein *via* the proteasome^12^. In human tumors, the PI3K/AKT/mTOR cellular pathway is frequently found over activated leading therefore to inhibition of the transcriptional activity of the FOXO proteins^5,13^.

Various studies strongly suggest that FOXO proteins are tumor suppressors. The conditional deletion of all *FOXO1*/*3*/4 alleles in adult mouse tissues induces the development of lymphoblastic thymic lymphomas and hemangiomas^14^. Overexpression of FOXO1 and FOXO3 proteins in breast cancer has been shown to inhibit the growth of breast cancer cells^15–18^. IκB kinase and ERk promote breast carcinogenesis *via* inhibition of FOXO3 protein expression [9]. The cytoplasmic expression of the FOXO3 protein is positively correlated with poor survival in breast cancer^15^. Low expression of FOXO1 or FOXO3 protein in breast tumors is correlated with poor clinical outcome^19,20^. Altogether, these results strongly suggest that FOXO1 and FOXO3 proteins act as tumor suppressors in breast cancer. However, other studies have described unexpected functions for these two FOXO proteins in resistance to breast cancer treatment and breast cancer promotion^21–24^. Notably, FOXO1 and FOXO3 proteins have been implicated in the promotion of breast tumor cell invasion^21,22^. FOXO3 protein expression has also been associated with poor survival in breast cancer^23,24^.

In order to better define the role of FOXO1 and FOXO3 proteins in breast cancer, we performed a comparative study of their RNA and protein expressions in 218 breast tumors by using real-time quantitative reverse-transcription polymerase chain reaction (qRT-PCR) and Reverse Phase Protein Arrays (RPPA) methods, respectively. We also determined the correlations between FOXO1 and FOXO3 protein expressions and classical clinical biological parameters, as well as the expression of proteins involved in the PI3K/AKT/mTOR pathway, DNA damage response (DDR), apoptosis, cell cycle, and/or cell proliferation.

## Results

### FOXO1 and FOXO3 RNA and protein expressions in breast cancer

To better define the role of *FOXO* genes in breast cancer, we analysed their RNA and protein expression in a series of 218 breast tumors (clinical parameters presented in Table S1).

In keeping with our previous work^25^, we found that the expressions of *FOXO1*, *FOXO*4, and *FOXO6* at the RNA level varied widely in breast tumors compared to normal breast samples (Table S2). In general, *FOXO1* and *FOXO4* mRNA were significantly underexpressed (expression values < 0.33), whereas *FOXO6* mRNA was overexpressed (expression values ≥ 3), compared to normal breast tissue (see Material and Methods page 11). *FOXO4* expression in the HR + ERBB2 + subtype and *FOXO6* expression in the HR- ERBB2- subtype were similar in tumors and normal breast tissue. *FOXO*3 gene expression was similar in tumors and normal breast tissue except in the *HR* + *ERBB2* + subtype, in which it was slightly, but significantly overexpressed.

The RPPA method demonstrated a wide range of FOXO1 protein expression (ranging from 0.07 to 1.91) and FOXO3 protein expression (ranging from 0.19 to 3.98) in breast tumors (Table 1). FOXO4 and FOXO6 protein expression was not studied due to the lack of an appropriate specific antibody for these two proteins.

**Table 1:** Protein levels of FOXO1 and FOXO3 in the series of 218 breast tumours.

	n=	FOXO1	FOXO3
*Total population*			
Median (range)	218	1.0 (0.07–1.91)	1.0 (0.19–3.98)
Low level (%)^a^		23 (10.6)	16 (7.3)
High level (%)^a^		0 (0)	13 (6.0)
*HR− ERBB2−*			
Median (range)	44	1.03 (0.11–1.91)	0.84 (0.39–2.52)
Low level (%)^a^		3 (6.8)	4 (9.1)
High level (%)^a^		0 (0)	1 (2.3)
*HR- ERBB2*+			
Median (range)	42	1.11 (0.46–1.81)	0.98 (0.41–2.77)
Low level (%)^a^		1 (2.4)	2 (4.8)
High level (%)^a^		0 (0)	3 (7.1)
*HR* + *ERBB2−*			
Median (range)	112	0.95 (0.12–1.76)	1.08 (0.19–3.98)
Low level (%)^a^		16 (14.3)	9 (8.0)
High level (%)^a^		0 (0)	8 (7.1)
*HR* + *ERBB2* +			
Median (range)	20	1.03 (0.07–1.66)	1.00 (0.45–2.67)
Low level (%)^a^		3 (15.0)	1 (5.0)
High level (%)^a^		0 (0)	1 (5.0)

Protein levels were normalized so that the median of values in the 218 breast tumours was 1.

^a^Low and high protein levels were defined as twofold variations of level, relative to the median level of the series of 218 breast tumors.

**Figure 1:**
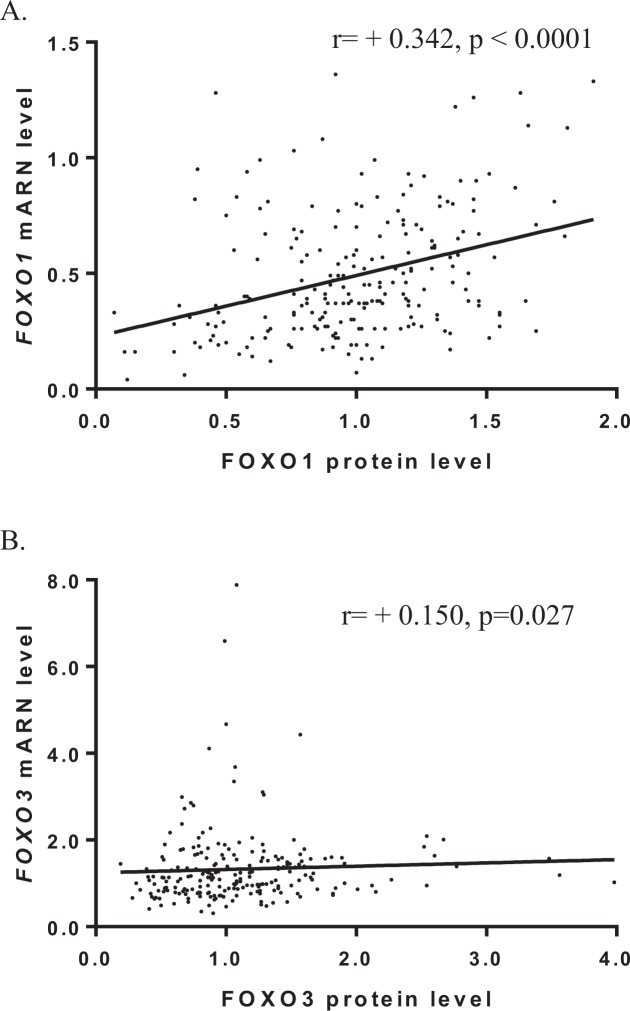
Scatter plots and Spearman correlation coefficients (r) between *FOXO1* (**A**) and *FOXO3* (**B**) protein and mRNA levels in a series of 218 breast tumors.

We examined the correlation between protein and mRNA expressions for *FOXO1* and FOXO*3* genes. We observed weak positive correlation for the *FOXO1* gene (r = +0.342, *p* < 10^−4^) and a weak positive correlation for the *FOXO3* gene (r = +0.150, *p* = 0.027), strongly suggesting that the expression of these two genes in breast tumors is regulated by molecular mechanisms independent of transcription and RNA stability (Fig. 1). These results also highlight the importance of considering protein expression when studying the role of *FOXO1* and *FOXO3* genes in breast cancer.

### Relationship between FOXO1 and FOXO3 protein expressions and clinical biological parameters of breast cancer

We investigated the relationships between FOXO1 and FOXO3 protein expressions and several classical clinical biological parameters (Tables 2 and 3). Marked differences were observed between these two FOXO proteins, as high FOXO1 protein expression was associated with negative estrogen receptor α (ERα) and progesterone receptor (PR) status, while low FOXO3 protein expression was weakly associated with these two biological parameters. We also showed that high FOXO3 protein expression was associated with low SBR histological grade, and, surprisingly, with high level of lymph node status. However, no association was observed between FOXO1 protein expression and these two clinical parameters.

**Table 2:** Relationship between FOXO1 protein levels and classical clinical biological parameters in the series of 218 breast tumours.

	Total population (%)	FOXO1 protein levels	*p*-value^a^
*Total*	218 (100)	1.0 (0.07–1.91)	
*Age*			
≤50	54 (24.8)	1.02 (0.32–1.81)	0.71 (NS)
>50	164 (75.2)	0.99 (0.07–1.91)	
*SBR histological grade*^b,c^			
I	22 (10.4)	0.93 (0.43–1.66)	0.21 (NS)
II	86 (40.8)	0.99 (0.07–1.65)	
III	103 (48.8)	1.03 (0.11–1.91)	
*Lymph node status*^d^			
0	69 (31.9)	1.00 (0.11–1.69)	0.77 (NS)
1–3	84 (38.9)	0.98 (0.07–1.81)	
>3	63 (29.2)	1.01 (0.15–1.91)	
*Macroscopic tumor size*^e^			
≤25 mm	82 (38.5)	0.99 (0.11–1.80)	0.97 (NS)
>25 mm	131 (61.5)	1.00 (0.07–1.91)	
*ERα status*			
Negative	89 (40.8)	1.06 (0.11–1.91)	**0.0047**
Positive	129 (59.2)	0.95 (0.07–1.76)	
*PR status*			
Negative	117 (53.7)	1.03 (0.07–1.91)	**0.0037**
Positive	101 (46.3)	0.95 (0.15–1.76)	
*ERBB2 status*			
Negative	156 (71.6)	0.98 (0.11–1.91)	**0.071**
Positive	62 (28.4)	1.09 (0.07–1.81)	
*Molecular subtypes*			
HR- ERBB2−	44 (20.2)	1.03 (0.11–1.91)	**0.022**
HR− ERBB2+	42 (19.3)	1.11 (0.46–1.81)	
HR + ERBB2−	112 (51.4)	0.95 (0.12–1.76)	
HR + ERBB2+	20 (9.2)	1.03 (0.07–1.66)	

NS: not significant. ^a^Mann-Whitney (2 groups) or Kruskal Wallis (more than 2 groups) test. ^b^Scarff Bloom Richardson classification. ^c^Information available for 211 patients. ^d^Information available for 216 patients. ^e^Information available for 213 patients.

**Table 3:** Relationship between FOXO3 protein levels and classical clinical biological parameters in the series of 218 breast tumours.

	Total population (%)	FOXO3 protein levels	*p*-value^a^
*Total*	218 (100)	1.0 (0.19–3.98)	
*Age*			
≤50	54 (24.8)	1.06 (0.39–2.27)	0.77 (NS)
>50	164 (75.2)	0.99 (0.19–3.98)	
*SBR histological grade*^b,c^			
I	22 (10.4)	1.33 (0.43–3.56)	**0.010**
II	86 (40.8)	1.02 (0.19–3.98)	
III	103 (48.8)	0.91 (0.28–3.48)	
*Lymph node status*^d^			
0	69 (31.9)	0.87 (0.19–2.77)	**0.0010**
1–3	84 (38.9)	1.01 (0.28–3.98)	
>3	63 (29.2)	1.08 (0.31–2.67)	
*Macroscopic tumor size*^e^			
≤25 mm	82 (38.5)	1.07 (0.28–3.48)	0.22 (NS)
>25 mm	131 (61.5)	0.98 (0.19–3.98)	
*ERα status*			
Negative	89 (40.8)	0.91 (0.39–2.77)	**0.029**
Positive	129 (59.2)	1.08 (0.19–3.98)	
*PR status*			
Negative	117 (53.7)	0.94 (0.31–2.77)	**0.013**
Positive	101 (46.3)	1.08 (0.19–3.98)	
*ERBB2 status*			
Negative	156 (71.6)	1.02 (0.19–3.98)	0.89 (NS)
Positive	62 (28.4)	0.99 (0.41–2.77)	
*Molecular subtypes*			
HR− ERBB2−	44 (20.2)	0.84 (0.39–2.52)	**0.044**
HR− ERBB2+	42 (19.3)	0.98 (0.41–2.77)	
HR+ ERBB2−	112 (51.4)	1.08 (0.19–3.98)	
HR+ ERBB2+	20 (9.2)	1.00 (0.45–2.67)	

NS: not significant. ^a^Mann-Whitney (2 groups) or Kruskal Wallis (more than 2 groups) test. ^b^Scarff Bloom Richardson classification. ^c^Information available for 211 patients. ^d^Information available for 216 patients. ^e^Information available for 213 patients.

**Figure 2:**
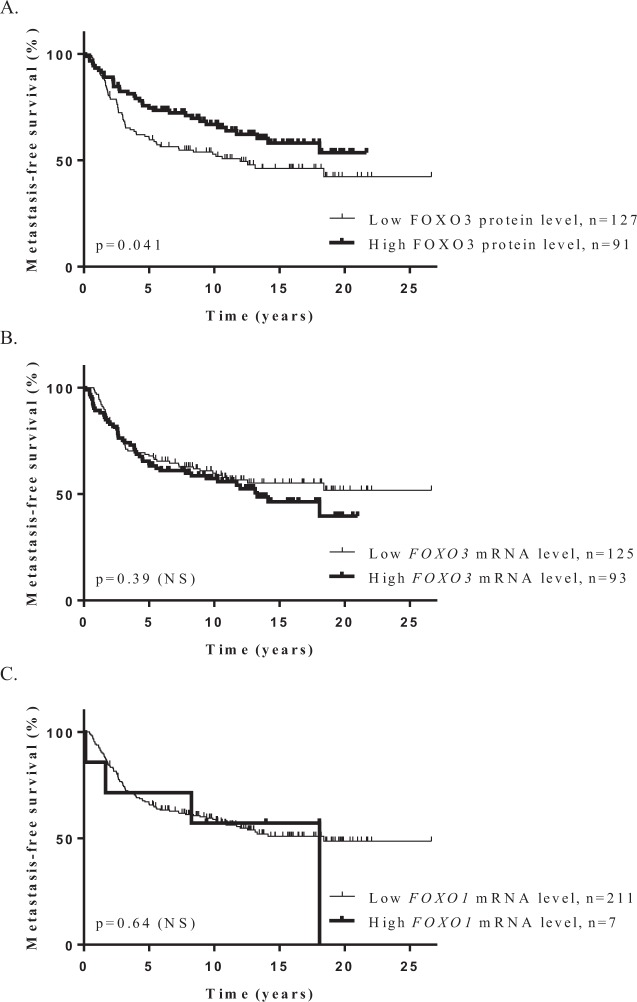
Kaplan-Meier metastasis-free survival curves for *FOXO3* and *FOXO1* genes, according to protein levels (**A**) for FOXO3 and mRNA levels (**B,C**) for *FOXO1* and *FOXO3* in a series of 218 breast tumors. P-values are estimated using the log-rank test. Patients with breast tumors expressing high levels of FOXO3 protein had significantly better MFS than patients with breast tumors expressing lower levels of this protein (*p* = 4.1.10^−2^) (**A**). *FOXO3* and *FOXO1* mRNA expressions have no prognostic value (**B** and **C** respectively).

To investigate in more detail the role of *FOXO1* and *FOXO3* genes in breast cancer, we also performed a log rank test to analyse the relationship between FOXO1 and FOXO3 protein expressions and metastasis-free survival (MFS). Patients with breast tumors expressing high levels of FOXO3 protein had better MFS than patients with breast tumors expressing lower levels of FOXO3 protein (*p* = 4.1.10^−2^), which is consistent with a tumor suppressor role (Fig. 2A). Such an association was not observed for FOXO1 protein. Multivariate analysis using a Cox proportional hazards model assessed the predictive value for MFS of the parameters found to be significant in univariate analysis (Table S1), i.e. lymph node status and macroscopic tumor size, and FOXO3 protein expression (*p* = 5.5.10^−3^) (Table S3). The prognostic significance of these three parameters persisted in multivariate analysis, indicating that FOXO3 protein expression is an independent prognostic factor in breast cancer. Interestingly, no correlation was demonstrated between *FOXO1* or *FOXO3* mRNA expression and MFS in this series of 218 breast tumors (Fig. 2B,C), highlighting once again the importance of studying FOXO1 and FOXO3 protein expressions in order to assess their role in breast cancer.

Altogether, these observations suggest that FOXO3 protein, but not FOXO1 protein, may act as a tumor suppressor in breast cancer.

### Relationship between the levels of FOXO1, FOXO3, and other proteins involved in the PI3K/AKT/mTOR pathway, DDR, apoptosis, cell cycle, and cell proliferation

In order to better define the role of FOXO1 and FOXO3 proteins in breast cancer, we also used the RPPA approach to study other proteins involved in the PI3K/AKT/mTOR oncogenic pathway, which regulates the activities of FOXO proteins, as well as various cellular processes involved in cancer and regulated by FOXO proteins: DDR, apoptosis, cell cycle, and cell proliferation (Table 4).

**Table 4:** Spearman rank correlation coefficients (r) and *p*-values between FOXO1 and FOXO3 protein levels and other proteins of different pathways in the series of 218 breast tumours.

	FOXO1 protein		FOXO3 protein	
	r	*p*-value	r	*p*-value
**PI3K/AKT/mTOR pathway**				
Phospho-S6K (Thr-421/Ser-424)		NS	−0.252	0.0002
Phospho-S6K (Thr-389)		NS	−0.163	0.016
Phospho-S6 (Ser-235/236)		NS	−0.186	0.0058
Phospho-S6 (Ser-24)	+0.141	0.038	−0.301	<0.0001
AKT		NS	−0.317	<0.0001
PDK1	−0.186	0.0060	−0.181	0.0073
mTor		NS	−0.178	0.0086
S6 Rib		NS	−0.446	<0.0001
IRS1		NS	−0.172	0.011
PTEN		NS		NS
**DNA repair**				
Ku80	−0.137	0.043	−0.327	<0.0001
DNA-PK		NS	−0.144	0.034
PARP		NS	−0.401	<0.0001
NBS1	−0.156	0.021	−0.303	<0.0001
RAD50		NS	−0.181	0.0072
Mre11	−0.137	0.043	−0.203	0.0026
**Apoptosis**				
P53		NS	+ 0.299	<0.0001
Phospho-p53 (Ser-15)		NS	+ 0.320	<0.0001
Phospho-p53 (Ser-392)		NS	+ 0.387	<0.0001
**Cell cycle**				
p15		NS	+ 0.392	<0.0001
**Proliferation**				
KI67	+ 0.134	0.049	−0.46	<0.0001

To evaluate the PI3K/AKT/mTOR pathway activity in our tumors, we studied the phosphorylation status of the S6K and S6, two proteins specifically phosphorylated by this pathway^26^. The PI3K/AKT/mTOR pathway-dependent phosphorylation of AKT to its threonine 308 (pAKT-T308) and, to a lesser extent, its serine 473, is essential for its kinase activity^27^. However, because of the lack of appropriate pAKT-T308 antibody for RPPA method, we did not use the phosphorylated forms of AKT to evaluate the PI3K/AKT/mTOR pathway activity. We found negative correlations between FOXO3 protein expression and protein expressions of Phospho-S6K (Thr-421/Ser-424) (r = −0.252, *p* = 2.10^−4^), Phospho-S6K (Thr-389) (r = −0.163, *p* = 1.6.10^−2^), Phospho-S6 (Ser-235/Ser-236) (r = −0.186, *p* = 5.8.10^−3^), and Phospho-S6 (Ser-24) (r = −0.301, *p* < 10^−4^), suggesting that the low expression of FOXO3 protein is associated to a weak activity of the PI3K/AKT/mTOR pathway in breast tumors. We observed that FOXO3 protein expression was also negatively correlated with the protein expressions of various components of this pathway: AKT (r = −0.317, *p* < 10^−4^), PDK1 (r = −0.181, *p* = 7.3.10^−3^), mTOR (r = −0.178, *p* = 8.6.10^−3^), S6 (r = −0.446, *p* < 10^−4^), and IRS1 (r = −0.172, *p* = 1.1.10^−2^)^28,29^. We did not detect significant correlation between FOXO3 protein expression and the protein expression of one of the most important negative regulators of the PI3K/AKT/mTOR pathway, PTEN. The weak PI3K/AKT/mTOR pathway activity found in the breast tumors expressing low level of FOXO3 protein would be therefore due to a low level of various components of this pathway but not to a high level of PTEN. Regarding FOXO1, we detected a negative correlation only with PDK1 (r = −0.186, *p* = 6.10^−3^). Akt activation has been shown to promote degradation of FOXO3 protein by proteasomes^12^. Therefore, our results suggest that high activity of the PI3K/AKT/mTOR pathway in breast tumors would induce degradation of FOXO3 protein, but not FOXO1 protein.

Interestingly, we also demonstrated negative correlations between FOXO3 protein expression and the protein expression of various factors involved in different DNA repair mechanisms: Ku80 (r = −0.327, *p* < 10^−4^) and DNA-PK (r = −0.144, *p* = 3.4.10^−2^) involved in non-homologous end joining (NHEJ), PARP (r = −0.401, *p* < 10^−4^) crucial for alternative NHEJ and base excision repair, and the three components of the MRN complex: NBS1 (r = −0.303, *p* < 10^−4^), RAD50 (r = −0.181, *p* = 7.2.10^−3^), and Mre11 (r = −0.203, *p* = 2.6.10^−3^), described as a key multi-protein complex crucial for DNA repair by homologous recombination and NHEJ^30,31^. Only weakly significant negative correlations were demonstrated between FOXO1 protein expression and Ku80, NBS1, and Mre11 (0.05 > *p* > 0.01) (Table 4). Strong FOXO3 protein expression in breast tumor cells therefore appears to inhibit DDR, which would lead to accumulation of genetic alterations, thereby causing cell cycle arrest and/or p53-dependent apoptosis. Consistent with this hypothesis, we found that the FOXO3 protein level was negatively correlated with the KI67 protein level, a marker of proliferation (r = −0.460, *p* < 10^−4^), and was positively correlated with the cell cycle inhibitor p15 protein level (r = +0.392, *p* < 10^−4^), as well as the levels of p53 (r = +0.299, *p* < 10^−4^), Phospho-p53 (Ser-15) (r = +0.320, *p* < 10^−4^), and Phospho-p53 (Ser-392) (r = +0.387, *p* < 10^−4^) (phosphorylation of p53 at these two sites triggers its apoptotic activity^32^), whereas FOXO1 protein level was very slightly positively correlated with KI67 (r = +0.134, *p* = 4.9. 10^−2^) and not correlated with cell cycle and apoptosis protein expressions.

In order to check that FOXO3 is functional in breast tumors, we performed a western blot analysis to visualize Phospho-FOXO3 (pSer-253), the inactive form of FOXO3, of several breast tumors of our series^10^. We detected at least 8 samples with negative or low levels of phospho-FOXO3 (pSer-253) expression among 12 breast tumor samples expressing high levels of FOXO3, suggesting that this FOXO3 protein may be functional in a majority of these tumors (Fig. S1).

Overall, our results suggest that FOXO3 protein, but not FOXO1 protein, acts as a tumor suppressor in breast cancer, at least in part by DDR inhibition and subsequent induction of p53-dependent apoptosis. They also suggest that the antitumor effect of FOXO3 is abolished by high activity of the PI3K/AKT/mTOR pathway.

## Discussion

Many studies designed to examine the role of genes in carcinogenesis determine the correlations between their RNA expression and classical clinical biological parameters, survival, and the expression of others genes linked to cancer. However, due to post-transcriptional regulations, weak correlations are commonly observed between RNA expression and protein expression^33^. In our study, we found weak correlations between FOXO1 and FOXO3 RNA and protein expressions in breast cancer (Fig. 1). The absence of correlation between protein and mRNA expressions for *FOXO1* and *FOXO3* genes can be fully explain by the fact that these FOXO proteins undergo posttranslational modifications, such as acetylation, ubiquitination, and phosphorylation, modulating their subcellular localization and stability. To investigate the role of *FOXO1* and *FOXO3* genes in breast carcinogenesis, we therefore used the RPPA method to perform a comparative study of the protein expression of these two *FOXO* genes in a series of 218 breast tumors.

Our results strongly suggest that FOXO3 protein, but not FOXO1 protein, acts as a tumor suppressor in breast cancer. In particular, we found that patients with breast tumors expressing high levels of FOXO3 protein had better survival rates than patients with breast tumors expressing lower levels of this protein. We also showed that FOXO3 protein expression, but not FOXO1 protein expression, was negatively correlated with the expression of the KI67 marker of proliferation (Table 4). We recently showed that FOXO6 protein has an oncogenic effect in breast cancer^25^. Therefore, despite their homologies, the FOXO proteins appear to have different and specific effects on breast cancer development.

In line with the results of various studies, we provide experimental arguments suggesting a tumor suppressor activity of FOXO3 protein in breast cancer^15–17,20^. However, other studies have shown that high FOXO3 protein expression is associated with poor disease-free survival in TN breast cancer, and promotes proliferation, migration and invasion of TN breast cancer cell lines^22,23^. In addition, Sisci *et al*. reported that FOXO3 protein inhibits breast carcinogenesis in ERα-positive cells, and tends to promote breast carcinogenesis in ERα-negative cells^34^. The role of FOXO3 protein in breast carcinogenesis may therefore depend on the subtype of breast cancer and the stage of disease. Further protein expression studies based on larger series of breast cancers are necessary to determine the precise role of FOXO3 protein in the various subtypes of breast cancer.

Several studies suggest that FOXO3 protein acts as a tumor suppressor in breast cancer by inducing the expression of cyclin-dependent kinase inhibitors (CDK inhibitors) and proapoptotic proteins^16,35^. In line with these findings, we showed that FOXO3 protein expression was positively correlated with expression of the p15 CDK inhibitor, p53 and two active forms of p53 phosphorylated at position S15 and S392 (Table 4). Surprisingly, we also demonstrated negative correlations between FOXO3 protein expression and the expression of numerous proteins involved in various DNA repair mechanisms, suggesting that high FOXO3 protein expression in breast tumors impairs DDR. Inhibition of DDR by FOXO3 protein could induce accumulation of DNA damage, thereby inducing p53-dependent apoptosis. FOXO3 was recently shown to negatively regulate the expression and activity of FOXM1, a forkhead protein activating the transcription of numerous genes involved in various DNA repair mechanisms and genotoxic agent resistance^36^. FOXO3 protein competes with FOXM1 for the binding to the same DNA motifs in target promoters and produces opposing transcriptional outputs. Therefore, one of the mechanisms by which FOXO3 protein could inhibit DDR in breast cancer, would be the inhibition of the transcription of DDR-genes induced by FOXM1.

PARP inhibitors have been shown to be highly lethal to tumor cells with a defect in DNA repair by homologous recombination called “BRCAness”. The activity of these inhibitors is based on the principle of synthetic lethality, which consists of targeting two separate molecular pathways that are nonlethal when disrupted individually, but are lethal when inhibited simultaneously. We found negative correlations between the expression of FOXO3 protein and that of the three components of the MRN complex (NBS1, RAD50, and Mre11) crucial for DNA repair by homologous recombination (Table 4). High expression of FOXO3 protein could therefore be an attractive predictive biomarker of favourable response to treatment with PARP inhibitors in breast tumors.

## Materials and Methods

### Patients and samples

The conditions of patient’s selection and sample collection were as previously described [19].

The treatment of the 218 patients (mean age: 61.3 years, range: 29–87 years) consisted of modified radical mastectomy in 140 cases (64.2%) and breast-conserving surgery plus locoregional radiotherapy in 77 cases (35.3%) (information available for 217 patients). 171 patients received adjuvant therapy: chemotherapy alone in 63 cases, hormone therapy alone in 75 cases and both treatments in 33 cases. The population was divided into four groups according to HR and ERBB2 status, as follows: two luminal subtypes (HR+ ERRB2+ (ER*α*+ and/or PR+, and ERBB2+, *n* = 20) and HR+ ERRB2 − (ER*α*+ and/or PR+, and ERBB2− , *n* = 112)); an ERBB2+ subtype (ER*α*− , PR− , and ERBB2+, *n* = 42)) and a triple-negative (TN) subtype (ER*α* − , PR − , and ERBB2 − , *n* = 44)). The median follow-up is 9.1 years (range 1 month to 27 years); 100 patients metastasized. Standard prognostic factors are shown in Table S1. The median follow-up was 9.1 years (range: 1 month to 27 years); 100 patients developed metastasis. Fifteen specimens of adjacent normal breast tissue from breast cancer patients or normal breast tissue from women undergoing cosmetic breast surgery were used as sources of normal mRNA^37^.

### Real-time qRT–PCR

The theoretical basis, RNA extraction, cDNA synthesis, design of primers and qRT-PCR conditions have been previously described in detail [33]. The *FOXO1* and *FOXO3* expression values of the samples were normalized such that the median value for the 15 normal breast tissues was 1. Variation in *FOXO1* and *FOXO3* expression values from one sample to another of the 15 normal breast are small (*FOXO1* ARNm median = 1.0, min = 0.51, max = 1.85. *FOXO3* ARNm median = 1.0, min = 0.71, max = 1.86.), indicating that these expressions are representative. The nucleotide sequences of the primers used were as follows: TBP-U (5′-TGCACAGGAGCCAAGAGTGAA-3′) and TBP-L (5′-CACATCACAGCTCCCCACCA-3′) for *TBP* gene (132 bp PCR product); FOXO1-U (5′-GTCAAGAGCGTGCCCTACTTCA-3′) and FOXO1-L (5′-TGAACTTGCTGTGTAGGGACAGATTAT-3′) for *FOXO1* gene (101 bp PCR product); FOXO3-U (5′-CCTACTTCAAGGATAAGGGCGACAG-3′) and FOXO3-L (5′-GTGCCGGATGGAGTTCTTCCAG-3′) for *FOXO3* gene (62 bp PCR product); FOXO4-U (5′-TGGTCCGTACTGTACCCTACTTCA-3′). Over- and under-expressions were defined as threefold variations of expression relative to the median expression of normal samples. We have previously used the same approach to determine cut-off points for tumor gene altered expression^38–40^.

### RPPA

RPPA technology was used for quantifying the relative abundance of total protein expression as previously described^41^. Antibody references are available in Table S4. Low and high protein expressions were defined as twofold variations of expression relative to the median expression of the series of 218 breast tumors.

### Western blot

Proteins from breast tumors were extracted with buffer A (50 mM Tris pH = 6.8, 2% SDS, 5% glycerol, 2 mM DTT, 2.5 mM EDTA, 2.5 mM EGTA, 4 mM sodium orthovanadate, 20 mM sodium fluoride, 1 mM PMSF). The antibodies used in this study were: anti-FOXO3 (9467, Cell signalling, Beverly, MA, USA), anti-Phospho-FOXO3A (pSer-253) (ab47285, abcam, Cambridge, MA), and anti-GAPDH used as internal control (sc-20357, Santa Cruz Biotechnology, Santa Cruz, CA). Proteins were detected by the ECL Western Blotting Analysis System procedure (GE Healthcare, Buckinghamshire, UK).

### Statistical analysis

Statistical analyses were done as previously described [19]. The Cox proportional hazards regression model was used to assess prognostic significance and the results are presented as hazard ratios (HR) and 95% confidence intervals (CIs).

### Compliance with ethical standards

All procedures performed in studies involving human participants were in accordance with the ethical standards of the institutional and/or national research committee and with the 1964 Helsinki declaration and its later amendments or comparable ethical standards. All patients who entered our institution before 2007 were informed that their tumor samples might be used for scientific purposes and were given the opportunity to decline. Since 2007, patients entering our institution have also provided their approval by signing an informed consent form. This study was approved by the local ethics committee (René Huguenin Hospital Breast Group). Informed consent: Informed consent was obtained from all individual participants included in the study.

## Supplementary information

Supplementary tables and figure.
