# Supplementary material for: The high protein expression of FOXO3, but not that of FOXO1, is associated with markers of good prognosis
Source: Sci Rep. 2020 Apr 24;10:6920. doi: 10.1038/s41598-020-63895-8 (PMC7181619; doi:10.1038/s41598-020-63895-8)
Supplement: Supplementary file 1 — Supplementary tables and figure. [file 41598_2020_63895_MOESM1_ESM.pdf]

The high protein expression of FOXO3, but not that of FOXO1, is associated with markers of good prognosis.

François Lallemand, Sophie Vacher, Leanne de Koning, Ambre Petitalot, Adrien Briaux, Keltouma Driouch, Céline Callens, Anne Schnitzler, Caroline Lecerf, Floriane Oulie-Bard, Aurélie Barbet, Anne Vincent, Sophie Zinn-Justin, Bernard S. Lopez, Rosette Lidereau, Ivan Bieche, and Sandrine M. Caputo.

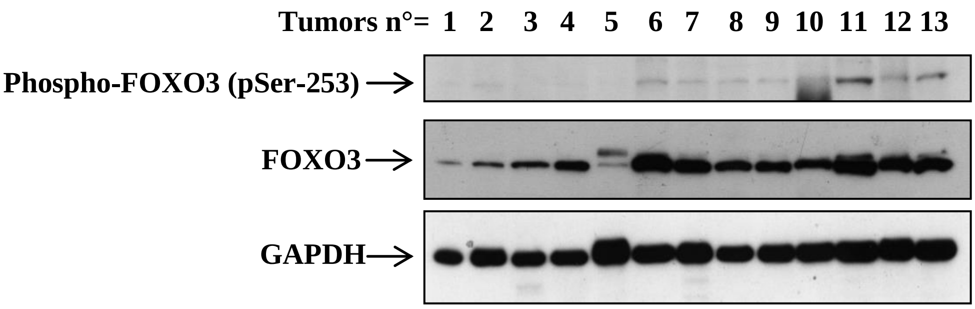

Figure S1: FOXO3 and Phospho-FOXO3 (ser-253) expressions in breast tumors. Breast tumor extracts were analysed by immunoblotting for their expressions of FOXO3, Phospho-FOXO3 (Ser-253), and GAPDH (loading control).

Table S1: Characteristics of the 218 breast tumours

	Number of patients (%)	Number with metastases (%)	*p*-value [a]
Total	218 (100)	100 (45.9)	
Age			
≤50	54 (24.8)	27 (50.0)	0.35 (NS)
>50	164 (75.2)	73 (44.5)	
SBR histological grade [b,c]			
I	22 (10.4)	7 (31.8)	0.22 (NS)
II	86 (40.8)	41 (47.7)	
III	103 (48.8)	51 (49.5)	
Lymph node status [d]			
0	69 (31.9)	20 (29.0)	**< 0.0001**
1-3	84 (38.9)	37 (44.0)	
>3	63 (29.2)	42 (66.7)	
Macroscopic tumor size [e]			
≤25mm	82 (38.5)	26 (31.7)	**0.0009**
>25mm	131 (61.5)	73 (55.7)	
ERα status			
Negative	89 (40.8)	42 (47.2)	0.34 (NS)
Positive	129 (59.2)	58 (45.0)	
PR status			
Negative	117 (53.7)	56 (47.9)	0.24 (NS)
Positive	101 (46.3)	44 (43.6)	
ERBB2 status			
Negative	156 (71.6)	68 (43.6)	0.18 (NS)
Positive	62 (28.4)	32 (51.6)	
Molecular subtypes			
HR- ERBB2-	44 (20.2)	17 (38.6)	0.12 (NS)
HR- ERBB2+	42 (19.3)	24 (57.1)	
HR+ ERBB2-	112 (51.4)	51 (45.5)	
HR+ ERBB2+	20 (9.2)	8 (40.0)	

NS: not significant.

[a] Log-rank test.

[b] Scarff Bloom Richardson classification.

[c] Information available for 211 patients.

[d] Information available for 216 patients.

[e] Information available for 213 patients.

Table S2: mRNA expression levels of *FOXO1*, *FOXO3*, *FOXO4* and *FOXO6* in normal breast samples and in the series of 218 breast tumours

	n=	*FOXO1*	*FOXO3*	*FOXO4*	*FOXO6*
Normals					
Median (range)	15	1.0 (0.51-1.85)	1.0 (0.71-1.86)	1.0 (0.44-1.49)	1.0 (0.36-2.34)
Under-expressed (%) [a]		0 (0)	0 (0)	0 (0)	0 (0)
Over-expressed (%) [a]		0 (0)	0 (0)	0 (0)	0 (0)
Total population					
Median (range)	218	0.41 (0.04-1.36)	1.14 (0.31-7.88)	0.59 (0.14-3.32)	1.77 (0.19-23.1)
Under-expressed (%) [a]		73 (33.5)	1 (0.5)	30 (13.8)	4 (1.8)
Over-expressed (%) [a]		0 (0)	9 (4.1)	1 (0.5)	51 (23.4)
p-value [b]		**< 0.0001**	0.55 (NS)	**0.0002**	**0.0004**
HR- ERBB2-					
Median (range)	44	0.45 (0.13-1.36)	1.26 (0.49-6.59)	0.56 (0.14-3.32)	1.29 (0.19-7.74)
Under-expressed (%) [a]		14 (31.8)	0 (0)	11 (25.0)	4 (9.1)
Over-expressed (%) [a]		0 (0)	4 (9.1)	1 (2.3)	7 (15.9)
p-value [b]		**< 0.0001**	0.12 (NS)	**0.0073**	0.18 (NS)
HR- ERBB2+					
Median (range)	42	0.55 (0.07-1.28)	1.17 (0.41-4.67)	0.54 (0.15-2.90)	1.75 (0.47-11.1)
Under-expressed (%) [a]		11 (26.2)	0 (0)	7 (16.7)	0 (0)
Over-expressed (%) [a]		0 (0)	3 (7.1)	0 (0)	9 (21.4)
p-value [b]		**0.0002**	0.36 (NS)	**0.0002**	**0.0005**
HR+ ERBB2-					
Median (range)	112	0.38 (0.04-1.28)	1.01 (0.31-4.43)	0.58 (0.14-1.81)	1.82 (0.38-18.4)
Under-expressed (%) [a]		43 (38.4)	1 (0.9)	10 (8.9)	0 (0)
Over-expressed (%) [a]		0 (0)	1 (0.9)	0 (0)	28 (25.0)
p-value [b]		**< 0.0001**	0.67 (NS)	**< 0.0001**	**< 0.0001**
HR+ ERBB2+					
Median (range)	20	0.46 (0.18-1.14)	1.39 (0.78-7.88)	0.86 (0.20-2.45)	2.43 (0.62-23.1)
Under-expressed (%) [a]		5 (25.0)	0 (0)	2 (10.0)	0 (0)
Over-expressed (%) [a]		0 (0)	1 (5.0)	0 (0)	7 (35.0)
p-value [b]		**0.0001**	**0.015**	0.49 (NS)	**0.0001**

mRNA expression levels were normalized so that the median of values for normal breast tissues (n=15) was 1. [a] Over- and under-expressions were defined as threefold variations of expression relative to the median expression of normal samples. [b] Mann-Whitney test relative to normals.

Supplemental Table S3: Multivariate COX analysis of MFS for FOXO3 protein level in the series of 218 breast tumours

Characteristics		HR[a]	95% CI [b]	*p*-value [c]
Lymph node status	0	1		**< 0.0001**
	1-3	1.75	1.34-2.28	
	> 3	3.07	1.81-5.21	
Macroscopic tumor size	≤ 25mm	1		**0.0037**
	> 25mm	1.97	1.24-3.11	
FOXO3 protein level	Low	1		**0.0055**
	High	0.55	0.36-0.84	

[a] Hazard ratio.
[b] 95% Confidential Interval.
[c] Multivariate COX analysis.

Supplemental Table S4: Antibodies used in this study

**Antibody**	**From**	**Ref.**
Phospho-S6K (Thr-421/Ser-424)	Upstate (Millipore)	04-393
Phospho-S6K (Thr-389)	Upstate (Millipoe)	04-392
Phospho-S6 Rib (Ser-235/236)	CST	2211
Phospho-S6 Rib (Ser-240/244)	CST	2215
AKT	CST	9272
PDK1	CST	5662
mTor	Abcam	Ab51089
S6 Rib	CST	2212
IRS1	Upstate (Millipoe)	06-248
Ku80	CST	2180
DNA.PK	Upstate (Millipoe)	05-907
PARP	Epitomics	1077-1
NBS1	CST	3002
RAD50	CST	3427
Mre11	CST	4847
p15	CST	4822
p53	CST	9282
Phospho-p53 (Ser-15)	CST	9286
Phospho-p53 (Ser-392)	Epitomics	2326-1
KI67	Dako	M7240
FOXO1	CST	2880
FOXO3	CST	9467
